# Orthodontic-surgical treatment: neuromuscular evaluation in open and deep skeletal bite patients

**DOI:** 10.1186/2196-1042-14-41

**Published:** 2013-10-29

**Authors:** Giampietro Farronato, Lucia Giannini, Guido Galbiati, Santo Andrea Stabilini, Cinzia Maspero

**Affiliations:** Fondazione IRCCS Cà Granda, Department of Orthodontics, University of Milan, 20100 Milan, Italy

## Abstract

**Background:**

The aim of this study was to compare electromyographic data of two groups of patients (open and deep skeletal bite) before and after surgical orthodontic treatment.

**Methods:**

All patients who underwent orthognathic surgery at the Department of Orthodontics (University of Milan) were subjected to periodic electromyographic evaluation of the masticatory muscles (masseter and anterior temporal muscles) and to electrokinesiographic evaluation of mandibular movements. The sample comprised 72 patients (35 open skeletal bite patients and 37 deep skeletal bite patients) at the end of craniofacial growth. The electromyographic instruments used in the study included a Freely and a K6-I electromyograph. Statistical evaluation was carried out with Student’s *t* tests for independent samples.

**Results:**

Lots of differences between open and deep skeletal bite patients have been underlined by the analysis of the electromyographic data obtained. These results have been obtained with both electromyographic systems. Muscular activity in microvolts is higher in deep skeletal bite patients at the beginning of the treatment than in open bite ones, but during the following phases of the treatment, the two values became similar.

**Conclusions:**

Morphologic differences between open and deep bite patients can also be demonstrated by instrumental examinations, and their correction after surgical treatment is observable on electromyographic and electrognatographic exams.

## Background

Facial growth and craniofacial morphology are influenced by both genetic and extrinsic factors [1–4]. Alterations in growth intensity and in soft tissue and muscle function can influence individual dentoalveolar development and the evolution of vertical malocclusion [5]. Clinicians routinely measure the upper and lower face height and the vertical jaw relationship, but it is also important to consider dentoalveolar compensation [6–9].

Besides, a study of Tausche et al. [7] showed that dental vertical malocclusions are very frequent: an anterior open bite was registered in 17% while deep bite more than 3.5 mm affected 46% of a group of 8,768 children aged between 6 and 17 years chosen by Tausche and colleagues for a study on the prevalence of malocclusions in the early mixed dentition. Lots of authors studied the relationship between temporomandibular joint health, masticatory muscle function, and craniofacial morphology, but only few authors investigated neuromuscular activity in open and deep bite patients before and after orthognathic surgery [10–19].

The pattern of resting electromyographic (EMG) activity in relation to skeletal sagittal and vertical facial types has been discussed. Antonini et al. [20] and Miralles et al. [21] reported opposite results about the connections between alterations on sagittal plane and neuromuscular function.

Controversy among authors about the relationship between masticatory muscle activity and craniofacial morphology seems to be due to differences in criteria about patient selection. For the vertical aspect, Ahlgren et al. [22, 23] and Lowe et al. [24] obtained different results about the correlation of craniofacial morphology and resting EMG activity.

Ahlgren et al. [22, 23] proved that the mandibular plane angle (SN-GoMe) was positively correlated to temporal muscle activity, while Lowe et al. [24] obtained contradictory results. Moller [25], Ingervall and Thilander [26], Bakke and Michler [27], Kayukawa [28], Farronato et al. [29], and Bong et al. [30] demonstrated that high mandibular angle cases were associated with weaker musculature as opposed to stronger musculature in the low-angle cases. Kayukawa [28] and Farronato et al. [29] showed higher masticatory muscle activities in deep bite patients than in open bite ones.

Ueda et al. [1] suggested that masseter muscle activity showed significant negative correlations with vertical craniofacial morphology, whereas temporal muscle activity was positively correlated. Tanaka et al. [31] and Aknin [32] suggested that most patients with open bite and deep bite showed masticatory muscle dysfunction. The vertical dimension of the face, as well as the proclination of the incisors, appears to affect lower lip function, which has a higher activity in deep bite patients [33, 34].

The aim of orthodontic-surgical treatment consists of repositioning the skeletal bone basis in a normal position in subjects where its position was not correct at the end of craniofacial growth. In literature, there is controversy about the use of mandibular surgery to correct anterior deep and open bite.

Burden et al. [35] and Swinnen et al. [36] showed that mandibular surgery is associated with poor outcomes while another study proved relatively good clinical dental and skeletal stability 1 year postsurgery in open bite patients treated by posterior Le Fort I impaction as well as with anterior extrusion. Orthodontic-surgical treatment has consequences on all masticatory muscles; however, the majority of studies in literature referred to the masseter and temporal muscles: they are the most accessible for the methodology analysis [37–39].

Lots of studies in literature have tried to investigate orthognathic surgery’s consequences on the neuromuscular system, but only few authors investigated neuromuscular activity in open and deep bite patients before and after orthognatic surgery. During the presurgical orthodontic phase, there is a reduction of maximum bite force and mandibular excursion [4, 40–44].

The modifications consequent to a surgical reposition of the bone basis present a huge variability due to the different sensibilities of patients on dental, muscular, and articulation levels. [45]. The majority of studies have analyzed only a single phase of the treatment. The aim of this study was to compare electromyographic data of two groups of patients (open and deep skeletal bite) before and after surgical orthodontic treatment.

## Methods

### Study group

This study included 72 Caucasic patients (35 open skeletal bite patients and 37 deep skeletal bite patients, 46 females and 26 males) in orthodontic-surgical treatment periodically submitted to an electromyographic evaluation of the masticatory muscles and an electrokinesiographic evaluation of mandibular movements.

The selected sample was chosen among 94 consecutive patients using the following criteria: all of them had to attend all the EMG protocol. Vertical classification was conducted basing on the School of Milan protocol (SOR-SNA/SNA-ME distances were considered).

The three main criteria that had to be satisfied for inclusion of the patients were the following: adult age (≥18 years), presence of a dentoskeletal discrepancy, and the need for combined surgical orthodontic treatment. The choice of surgical treatment was related to every single case. Some of them were bimaxillary surgical operations, and others involved only one maxillary bone.

A control group has been later selected, composed of 14 Caucasic subjects (7 men and 7 women). These subjects have been selected because of adult age, skeletal class I, and the absence of dental and skeletal anomalies. They were enrolled from the School of Dental Hygiene of Milan, and they signed an informed consent to participate in the study. No ethical approval or ethical review board judgment was necessary because the electromyographic tests were not invasive.

### Electromyographic and kinesiographic analyses

Two electromyographic evaluations of the anterior temporal muscle and of the masseter muscle in its superficial component have been realized with two different electromyographic instruments. Moreover, a kinesiographic exam of mandibular movements has been made on every patient. The patient was always asked to answer if he/she was exempt from dental or skeletal pain that could cause some impediment in carrying out the exercises correctly. Also, the subjects of the control group have been submitted to electromyography with both machines and kinesiography.

The electromyographic instruments used were the electromyograph Freely (De Gotzen, Legnano, Italy) and the electromyograph K6-I EMG (Myotronics, Tukwila, WA, USA). Four (anterior temporal muscle and masseter muscle) among the eight channels of acquisition available have been used on both electromyographies.

The timing of the electromyographic and kinesiographic examinations that were performed on the surgical orthodontic patients included the following: during the first medical examination; after the beginning of the orthodontic therapy; during the presurgical phase of the bimonthly orthodontics; every month from 3 months before the surgical operation to 3 months after; the day before the surgical operation; during the intermaxillary fitting, to verify that there was no spasm of the muscles; at the removal of the fitting; during the postsurgical orthodontic phase, with the same times as those in the presurgical orthodontic phase; at the removal of the surgical bite; and during the follow-up.

The patients were examined in a specific, totally undisturbed, and noiseless room. They sat on a rigid stool with an adjustable height to provide an angle of 90° between the thigh and lower leg. The thighs were parallel to the floor, the back was upright, and the gaze was toward beyond the horizon. The head was in a natural head position [46].

After thorough cleansing of the skin of the face with a wad soaked in Neoxinal (0.5% clorhexidine in ≥70% alcohol) to reduce forehead impedance and to facilitate adhesiveness, the electrodes for the electromyographic acquisition were positioned on the patient. The electrode positions were the same for both electromyographic instruments and were not modified between the use of the instruments. The electrodes used were disposable. These bipolar electrodes were positioned according to the following procedure:

- *Masseter muscle*: From behind the seated subject, the operator palpated the muscle mass while the patient clenched the teeth. To position the bipolar electrode parallel to the muscle fibers, a line was drawn that connected the commissura labiorum oris with the tragum and another one drawn following the esocanto-goniac line. The position of the electrode was such that the superior pole lies at the intersection point between these two lines, with its major axis along the esocanto-goniac line.
- *Temporal muscle*: The muscle mass was palpated while the patient clenched the teeth, thus localizing the major axis of the zygomatic process of the frontal bone. The bipolar electrode was positioned along the line parallel to this process. In this way, the electrode was positioned parallel to the muscle fibers and positioned more or less superficially in comparison with the frontoparietal suture.

A grounding earth electrode was positioned on a silent muscular area of the forehead. According to the protocol, the electromyographic evaluation was carried out with the Freely instrument as the need for more time for the K6-I acquisition would run the risk of being too time-consuming and of making the patient feel more tired.

During this electromyographic evaluation, the patients made an arch clench in a maximum voluntary clench on cotton rolls positioned around the first molar for 5 s (the ‘cotton clench’) and an arch clench in a maximum voluntary clench without the interposed rolls for 5 s (the ‘clench’). This procedure followed that of the Sforza et al. protocol [46].

With the K6-1 electromyographic instrument, an analysis of the neuromuscular system at rest was carried out to provide an objective measurement of the electrical activity of the examined muscle at rest and to evaluate any eventual muscle hyperactivity, which is typical of temporomandibular dysfunction. Low levels of electric activity were analyzed during the maximum voluntary clench and the clench on cotton rolls (cotton clench) to evaluate the linear correlations between the electromyographic signal, recruit of the motor unit, and the force expressed in the isometric contraction.

The following indices were recorded and monitored: the temporal, masseter, and mean percentage overlapping coefficients (POCs), as the index of symmetrical distribution of the muscular activities; the asymmetry, activation, and torque coefficients; and the cotton clench, clench, and percentage impact. These electromyographic evaluations were carried out with the K6-1 instrument equipped with a ‘sensor cage’ fitted to the head of the patient and with the magnet positioned intraorally. The examination was for two electromyographic evaluations on the patients in the same position. The magnet used (Myotronics, Tukwila, WA, USA) was specifically made for the kinesiographic instrument used. To allow correct adhesiveness of the magnet, the bioadhesive Stomahesive was used (Myotronics, Tukwila, WA, USA).

For the mandibular kinesiography, the examination included the following: maximum opening, opening and closing speeds, maximum protrusion of the mandible on the anteroposterior plane, maximum right laterality, maximum left laterality, mandible rest position, centric occlusion, freeway space at rest, freeway space after transcutaneous electrical nerve stimulation (TENS), and distance between centric occlusion and maximum aperture.

After the kinesiographic analysis of the mandible, TENS was carried out, which relaxes the muscles innervated by the V and VII pairs of cranial nerves. TENS lasted 45 to 50 min in which two monopolar electrodes were used: Myotrode SG and Myomonitor J5 (Myotronics, Tukwila, WA, USA). A second trial was performed on the muscle at rest to determine the expected muscular electric activity reduction and the efficacy of the therapy.

As it has already been shown that the first functional examination of a subject is not necessarily reliable for a control group, the whole examination was repeated twice, taking into consideration only the values obtained in the second trial. Furthermore, it should be noted that whenever an electromyographic evaluation or a kinesiographic examination was carried out, each scan or electromyographic trial was repeated at least twice to determine its reliability and its repeatability. In cases of discrepancy, the same evaluation was performed again until repeatable results were obtained.

### Statistical tests

Data analysis was carried out with Student’s *t* test where the null hypothesis assumes that the differences between the groups were due to chance while according to the alternative hypothesis, the differences among the groups are real. Percentage values have been expressed in decimal for statistical comparison (e.g., 87% = 0.87). All the patients signed an informed consent to participate in the study.

## Results

**Figure 1:**
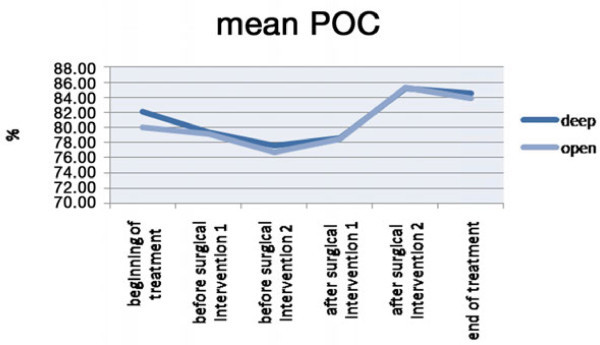
**Mean POC.**

The analysis of the data obtained from this work evidences differences among open bite patients and deep bite ones. POC values of the masseter muscle and the temporal muscle and the mean POC are slightly different among open and deep bite patients, but among these, only a few are statistically significant (POC of the temporal muscle and mean POC at the beginning of the treatment; Figure 1, Table 1).

**Table 1:** **Mean POC**

	Deep		Open	
	Mean	SD	Mean	SD
Beginning of the treatment	82.1	9.1	80	8.4
Before surgical intervention 1	79.5	7.2	79.2	8.9
Before surgical intervention 2	77.1	11.1	76.5	11.2
After surgical intervention 1	78.5	14.2	78.3	7.6
After surgical intervention 2	85.6	8.3	85.8	10.2
End of the treatment	84.4	8.9	84	7.9

Values are expressed in microvolts.

**Figure 2:**
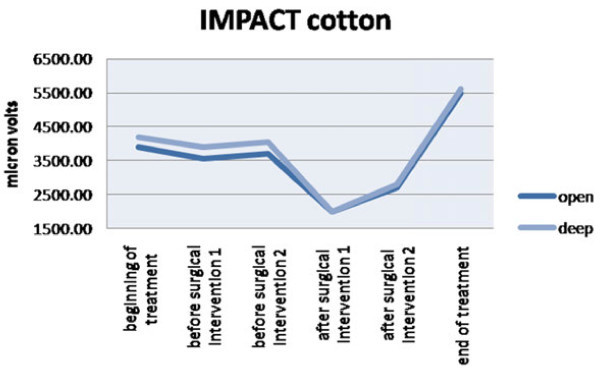
**The effect of the impact with the inclusion of the cotton rolls.**

**Figure 3:**
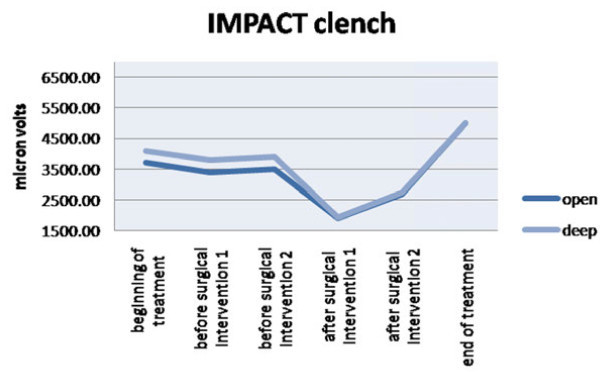
**The effect of the impact on the clench situation.**

Impact value, which evaluates muscular activity in time, is higher in deep bite patients than in open bite ones during treatment. At the end of the treatment, these values become not significantly different between the two groups (Figures 2 and 3, Tables 2 and 3). Muscular activity in microvolts is higher in deep skeletal bite patients at the beginning of the treatment than in open bite ones, but during the following phases of the treatment, the two values became similar.

**Table 2:** **Impact with cotton rolls**

	Deep		Open	
	Mean	SD	Mean	SD
Beginning of the treatment	4,258	451	3,971	380
Before surgical intervention 1	3,997	424	3,614	402
Before surgical intervention 2	4,100	583	3,702	390
After surgical intervention 1	2,100	284	2,082	367
After surgical intervention 2	2,702	250	2,641	300
End of the treatment	5,605	501	5,504	480

Values are expressed in microvolts.

**Table 3:** **Impact on clench**

	Deep		Open	
	Mean	SD	Mean	SD
Beginning of the treatment	4,087	512	3,702	432
Before surgical intervention 1	3,798	400	3,487	567
Before surgical intervention 2	3,990	528	3,569	378
After surgical intervention 1	2,071	322	1,965	345
After surgical intervention 2	2,670	201	2,592	254
End of the treatment	5,123	387	5,127	429

Values are expressed in microvolts.

This feature is evident on clenching on cotton rolls and on clenching the teeth. All these differences were statistically significant. Among mandibular protrusion movements, no one value was statistically different among open bite and deep bite patients while mandibular maximum opening was higher in deep bite patients in all the phases before surgical intervention, and this difference was statistically significant.

## Discussion

Lots of differences between open and deep skeletal bite patients have been underlined by the analysis of the electromyographic data obtained at the beginning of the treatment. The impact value and muscular activity in microvolt analysis shows a major muscle activity in deep bite patients than in open bite ones.

These results have been obtained with both electromyographic systems. The following authors also proved that high-angle cases were associated with weaker musculature than low-angle patients: Moller [25], Sassouni [47], Ingervall and Thilander [26], Bakke and Michler [27], Kayukawa [28], Farronato et al. [29], and Bong et al. [30].

Ahlgren et al. [22, 23] proved a positive correlation between the mandibular plane angle (SN-GoMe) and the temporal muscle activity (TMA). Moller [25] and Ingervall [48] obtained opposite results. Ueda et al. [1] proved that vertical craniofacial morphology is positively correlated with TMA and negatively correlated with masseter muscle activity (MMA). Fogle and Glaros [49] obtained opposite results. They proved that a correlation between craniofacial morphology and masticatory function does not exist. The only correlation is between muscle function and patient age.

The differences existing between the two groups at the beginning of the treatment, which is statistically significative, tend to disappear at the removal of the fixed orthodontic appliance, confirming the orthodontic-surgical treatment’s corrective role in accordance with Santoro and Maiorana’s study [50]. Furthermore, before the start of the fixed orthodontic therapy, patients present a compensatory equilibrium to malocclusion. During successive phases, electromyographic and electrognatographic values continue to worsen according to Thomas et al. [40], Brown and Moerenhout [51], Santoro and Maiorana [50], and Oliver and Knapman [52]. They improve in the postsurgical orthodontic phase only, during which it is useful to perform radiography to verify bone segment stability [53].

At the end of the orthodontic-surgical treatment, electromyographic values improve and reach optimal values similar to those of the control group. Mandibular movement rehabilitation needs more time than the muscular one even if it is satisfactory and constant too. At the end of the treatment, maximum mandibular opening is still less than the preoperative one. No statistically significative differences between the two groups have been highlighted about mandibular kinesiology.

## Conclusions

The Freely and K6-I instruments are different surface electromyographic instruments, projected and constructed in different ways. It is not possible to compare them, but data obtained from both instruments gives the clinicians the same information, even if they are expressed in two different ways. Results obtained from both electromyographic instruments show that functional rehabilitation in patients undergoing orthodontic-surgical treatment occurs in a good way and in a good time. Postsurgical rehabilitation presents a large interindividual variability, as suggested in literature [4, 45]. Moreover, a serious alteration of facial morphology does not always cause significative functional consequences.

The electromyographic evaluation of the masticatory muscles and the electrokinesiographic evaluation of mandibular movements during orthodontic-surgical therapy help the clinician reduce as much as possible an incorrect neuromuscular activity that can cause a relapse; it is also an important element to follow treatment phases and to control the results obtained. Morphologic differences between open and deep bite patients can also be demonstrated by instrumental examinations, and their correction after surgical treatment is observable on electromyographic and electrognatographic exams.

Through only the neuromuscular system and mandibular kinesiology, it is possible to obtain a successful steady result. At the end of this study, it is possible to state that a serious alteration of facial morphology is not always accompanied by functional consequences, and if this happens, it does not always involve all the parameters that determine correct neuromuscular function and mandibular kinesiology.
